# Healthy living, well-being and the sustainable development goals

**DOI:** 10.2471/BLT.18.222042

**Published:** 2018-09-01

**Authors:** Amina J Mohammed, Tedros Adhanom Ghebreyesus

**Affiliations:** a United Nations, New York, United States of America.; b World Health Organization, avenue Appia 20, 1211 Geneva 27, Switzerland.

Our world has made incredible progress since 2000 against several of the leading causes of illness and death. Life expectancy has increased,^1^ infant and maternal mortality have declined,^2^^,^^3^ malaria deaths have more than halved,^4^ and significant progress has been made against the human immunodeficiency virus epidemic.^5^

However, this progress has been fragile and uneven, both between and within countries. There remains a 31 year discrepancy between the countries with the shortest and longest life expectancies.^6^ While some countries have made impressive health gains, national averages can hide in-country disparities in health outcomes,^7^ for example in marginalized populations.

Meanwhile, increased prosperity around the world, changes in diets and lifestyles and rapid and unplanned urbanization have brought new health threats. The incidence of chronic respiratory diseases, diabetes, various types of cancer and road traffic injuries are all on the rise. Noncommunicable diseases account for some 70% of premature deaths, predominantly in low- and middle-income countries.^8^

The United Nations’ *Transforming our world: the 2030 agenda for sustainable development* provides us with an opportunity to address these challenges.^9^ The agenda is an ambitious vision of the healthier, more prosperous, inclusive and resilient world we want to see by 2030. The accompanying sustainable development goals (SDGs) provide the blueprint for action and are relevant to all countries, rich and poor. The particular attention given by the 2030 agenda to “leave no-one behind” is a clarion call to focus on those at most risk of missing out on the health services they need and deserve. Integrated and indivisible, the SDGs build on the momentum and lessons of the past and call for bold partnership across sectors to deliver on our shared promises.

While SDG 3 is devoted to good health and well-being, health contributes to almost all the other goals. For example, universal health coverage (UHC) can help to reduce poverty (SDG 1) by protecting people from a major cause of financial hardship, and good health can fuel increased employment and economic growth (SDG 8). Strong health systems can also provide support against the social and economic consequences of outbreaks and other health emergencies. Better health, in turn, advances the other goals.

In May 2018, the Member States of the World Health Organization (WHO) approved WHO’s new General Programme of Work 2019–2023. The programme is based on the SDGs and is designed to help countries stay on track towards SDG 3 and the other health-related targets.^10^ Its three strategic priorities – universal health coverage, health security and improved health and well-being – encapsulate each of the health-related targets, and are accompanied by an impact framework to enable WHO to measure progress and remain focused on outcomes rather than on outputs. Crucially, achieving these health targets will depend on the realization of people-centred primary health-care services that emphasize healthy living and disease prevention in addition to providing safe, effective and quality treatment.

Achieving the SDG health targets will require new investment. A WHO study estimated that in 67 countries accounting for about 75% of the world’s population, an additional 3.9 trillion United States dollars will be needed between 2015 and 2030 to achieve the SDG health targets.^11^ The study also estimated that domestic expenditure could meet 85% of those costs.^11^ In most countries, better health and well-being for all is therefore not fundamentally an economic choice – it’s a political choice.

The good news is that political support is growing around the world for UHC and the SDGs. For example, in April 2018, the Chancellor of Germany, Angela Merkel, the President of Ghana, Nana Addo Dankwa Akufo-Addo, and the Prime Minister of Norway, Erna Solberg, wrote a joint letter asking WHO to lead the development of a “Global Action Plan for Healthy Living and Well-being for All.”^12^

The United Nation (UN) Secretary-General’s development system reform, adopted by Member States in May 2018, seeks to ensure a UN system capable of responding to country priorities and supporting the implementation of the SDGs in a more integrated way.

Too often, fragmentation, duplication and inefficiency have undermined progress. We must break out of the silo mentality and ensure that the work of the UN development system is larger than the sum of its parts. Global health actors must work more closely together in support of national priorities and of countries’ achievement of the health-related SDG targets.

Both of us have seen in Africa and across the world the advances that good health has made possible. As partners we are now pushing the UN to strengthen its support to governments and people. We are committed to helping to integrate activities, enhance governance and maximize country-level impact towards the achievement of SDG 3 and the other health-related targets.

This issue of the *Bulletin of the World Health Organization* is a welcome addition to the growing body of literature that is needed to inform the strategies, policies, legislation, regulations and standards that will move us all closer to a fairer, safer and healthier world for all.
